# Three-dimensional electron diffraction as a complementary technique to powder X-ray diffraction for phase identification and structure solution of powders

**DOI:** 10.1107/S2052252514028188

**Published:** 2015-02-10

**Authors:** Yifeng Yun, Xiaodong Zou, Sven Hovmöller, Wei Wan

**Affiliations:** a Berzelii Center EXSELENT on Porous Materials and Inorganic and Structural Chemistry, Department of Materials and Environmental Chemistry, Stockholm University, SE-10691 Stockholm, Sweden

**Keywords:** three-dimensional electron diffraction, powder X-ray diffraction, phase identification, structure determination

## Abstract

Phase identification and structure determination are important and widely used techniques in chemistry, physics and materials science. Recently, two methods for automated three-dimensional electron diffraction (ED) data collection, namely automated diffraction tomography (ADT) and rotation electron diffraction (RED), have been developed. Compared with X-ray diffraction (XRD) and two-dimensional zonal ED, three-dimensional ED methods have many advantages in identifying phases and determining unknown structures. Almost complete three-dimensional ED data can be collected using the ADT and RED methods. Since each ED pattern is usually measured off the zone axes by three-dimensional ED methods, dynamic effects are much reduced compared with zonal ED patterns. Data collection is easy and fast, and can start at any arbitrary orientation of the crystal, which facilitates automation. Three-dimensional ED is a powerful technique for structure identification and structure solution from individual nano- or micron-sized particles, while powder X-ray diffraction (PXRD) provides information from all phases present in a sample. ED suffers from dynamic scattering, while PXRD data are kinematic. Three-dimensional ED methods and PXRD are complementary and their combinations are promising for studying multiphase samples and complicated crystal structures. Here, two three-dimensional ED methods, ADT and RED, are described. Examples are given of combinations of three-dimensional ED methods and PXRD for phase identification and structure determination over a large number of different materials, from Ni–Se–O–Cl crystals, zeolites, germanates, metal–organic frameworks and organic compounds to intermetallics with modulated structures. It is shown that three-dimensional ED is now as feasible as X-ray diffraction for phase identification and structure solution, but still needs further development in order to be as accurate as X-ray diffraction. It is expected that three-dimensional ED methods will become crucially important in the near future.

## Abstract 2

Two recently developed three-dimensional electron diffraction methods have shown great potential for phase identification and structure determination of polycrystalline powder materials. Their combination with powder X-ray diffraction makes them powerful techniques for phase identification in multiphase samples and the determination of very complex structures from nano- and micron-sized crystals.

## 1. Introduction   

Phase identification and structure determination of nano- and micron-sized crystals are important in materials science and crystallography. It is important to identify phases and to solve any new structures. The most widely used technique for phase identification is powder X-ray diffraction (PXRD). Each crystalline phase has its own unique PXRD pattern, providing a fingerprint for phase identification. Although PXRD is successful in many cases, a number of reasons limit the use of PXRD for multiphase samples, especially those containing unknown phases. A comparison of high-resolution transmission electron microscopy (HRTEM), electron diffraction (ED), single-crystal X-ray diffraction (SCXRD) and PXRD is given in Table 1 ▶.

X-ray diffraction (XRD) and electron crystallography are complementary techniques (Table 1 ▶). The most common technique for structure determination of crystalline materials is SCXRD, which can only be used for crystals larger than ∼10 µm with in-house diffractometers or a few microns with synchrotron light sources. The very strong interaction of electrons with matter makes electron crystallography amenable for studying crystals with sizes a million times smaller than what is needed for SCXRD. Crystals considered to be a powder when studied by XRD behave as single crystals in ED. While PXRD provides only one-dimensional information where diffraction peaks with similar *d* values overlap, ED provides three-dimensional information with no peak overlap. Determination of the unit-cell parameters and space group is straightforward from three-dimensional ED data, while it is sometimes difficult for PXRD, especially for structures with rather large unit-cell dimensions (≳10 Å). However, unit-cell parameters determined from ED are less accurate than those from PXRD. In addition, PXRD data are kinematic and complete, while ED data suffer from dynamic effects and are often incomplete. While PXRD data represent all phases present in the sample, three-dimensional ED provides information from individual particles. It is easy to select single crystals by ED for phase identification. The sizes of crystals suitable for ED studies range from nanometres to micrometres, depending on the type of material. However, the collection of three-dimensional ED data was previously very demanding and required expert experimentalists until the development of automated three-dimensional ED data collection and processing (Kolb *et al.*, 2007 ▶; Hovmöller, 2008 ▶). The phase information of the crystallographic structure factor, which is lost in diffraction, is present in high-resolution transmission electron microscopy (HRTEM) and high-resolution scanning transmission electron microscopy (STEM) images (Zou *et al.*, 1993*a* ▶; 1996 ▶; Weirich *et al.*, 1996 ▶; Willhammar, Mayoral & Zou, 2014 ▶). This is very useful for determining unknown structures by electron crystallography alone or in combination with PXRD.

PXRD is the most common technique for phase identification of crystalline samples, due to its simple and fast data collection and its well established databases, with powder patterns that serve as finger prints for phase identification. Although two-dimensional zonal ED patterns have also been used for phase identification, the collection and subsequent indexing of ED patterns are time consuming and require expertise (Zou *et al.*, 2004 ▶). However, ED has advantages in phase identification from multiphase samples, because individual particles can be selected within the TEM.

Both PXRD and electron crystallography have been used for the structure determination of nano- and micron-sized crystals. One major challenge of structure solution using PXRD data is handling overlapping reflections. Different methods have been developed to facilitate structure solution using powder diffraction data, for example direct methods (Altomare *et al.*, 1999 ▶, 2008 ▶) and charge-flipping (Baerlocher, McCusker & Palatinus, 2007 ▶). The zeolite-specific *FOCUS* method has also been developed by McCusker and co-workers that includes both crystal chemical information and powder diffraction data for structure solution of complex zeolites (Grosse-Kunstleve *et al.*, 1997 ▶). However, it is still difficult to solve complicated structures with a large unit cell by PXRD, when reflection overlap becomes severe. On the other hand, ED patterns facilitate better unit-cell parameters and space group determination because the reflections are separated as sharp spots in ED patterns. Although ED was used early on for structure determination (Vainshtein, 1964 ▶; Weirich *et al.*, 2000 ▶) and methods for quantifying ED patterns were developed (Zou *et al.*, 1993*a* ▶,*b* ▶), it was difficult and very demanding to obtain a large number of individual two-dimensional ED patterns along different zone axes and merge them into a three-dimensional data set. More importantly, ED intensities taken from zonal ED patterns suffer from dynamic effects. Precession electron diffraction (PED), invented by Vincent & Midgley (1994 ▶), provides higher resolution and is less affected by dynamic effects than conventional zonal ED (Oleynikov *et al.*, 2007 ▶). It has been used for solving the structures of unknown inorganic compounds from a projection or a series of PED zone-axis patterns (Gemmi *et al.*, 2003 ▶; Dorset *et al.*, 2007 ▶; Klein, 2011 ▶; Klein & David, 2011 ▶; Hadermann *et al.*, 2012 ▶).

As shown in Table 1 ▶, electron crystallography and PXRD are complementary to each other. The combination of electron crystallography and PXRD has been very powerful for structure determination of nano- and micron-sized crystals (McCusker & Baerlocher, 2009 ▶; Sun & Zou, 2010 ▶; Willhammar, Yun & Zou, 2014 ▶), especially using HRTEM images. The structure factor phases from HRTEM images were used to facilitate the solution of complex structures that could not be solved by PXRD alone, for example in the case of the zeolites TNU-9, IM-5 and SSZ-74, which were the three most complex zeolites at that point (Gramm *et al.*, 2006 ▶; Baerlocher, Gramm *et al.*, 2007 ▶; Baerlocher *et al.*, 2008 ▶). Zonal ED patterns have also been used in combination with PXRD for structure determination. Different strategies have been applied to combine ED with PXRD for structure determination of crystals. One strategy is to use structure factor phases retrieved from ED data as initial phases for structure determination from PXRD data. This was used for structure solution of the large-pore germanosilicate ITQ-26 (Dorset *et al.*, 2008 ▶). Xie and co-workers demonstrated how structure factor phases could be derived from two-dimensional PED patterns along four zone axes of zeolite ZSM-5 and used as the initial phase sets for powder charge flipping (Xie *et al.*, 2008 ▶). Another strategy is to use ED intensities for pre-partitioning of overlapping reflections in PXRD. The mesoporous chiral germanosilicate zeolite ITQ-37 was solved in such a way (Sun *et al.*, 2009 ▶).

Although electron crystallography, whether alone or in combination with PXRD, has been shown to be powerful for the determination of complex structures, structure determination has both been time-consuming (from months to years) and required extensive expertise (only a few groups could do it). Recent developments in automated three-dimensional ED data collection and processing (Kolb *et al.*, 2007 ▶, 2008 ▶; Hovmöller, 2008 ▶; Zhang *et al.*, 2010 ▶; Zou *et al.*, 2011 ▶; Wan *et al.*, 2013 ▶) have made both phase identification and structure solution faster and simpler, as feasible as SCXRD but from crystals a million times smaller than required by SCXRD. The combination of three-dimensional ED and PXRD is even more powerful for phase identification from multiphase samples and structure determination of nano- or micron-sized crystals (Kolb & Mugnaioli, 2011 ▶; Yun *et al.*, 2014 ▶). Three-dimensional ED has also been demonstrated on microcrystals of proteins and shown to be feasible for the structure solution of a few protein crystals (Shi *et al.*, 2013 ▶; Nannenga *et al.*, 2014 ▶; Nannenga & Gonen, 2014 ▶).

In this review, we present two newly developed three-dimensional ED methods, automated diffraction tomography (ADT) and rotation electron diffraction (RED), and their application as complementary techniques to PXRD for phase identification and structure determination. We show combinations of three-dimensional ED methods with PXRD on a large number of different materials, from Ni–Se–O–Cl crystals, zeolites, open-framework germanates, metal–organic frameworks and organic compounds to intermetallics with modulated structures.

## 2. The three-dimensional ED methods ADT and RED   

Two methods for automated three-dimensional ED data collection and processing were developed recently, namely automated diffraction tomography (ADT) (Kolb *et al.*, 2007 ▶, 2008 ▶) and rotation electron diffraction (RED) (Hovmöller, 2008 ▶; Zhang *et al.*, 2010 ▶; Zou *et al.*, 2011 ▶; Wan *et al.*, 2013 ▶). Both methods can be used for collecting almost complete three-dimensional ED data from nano- or micron-sized crystals.

**Figure 1:**
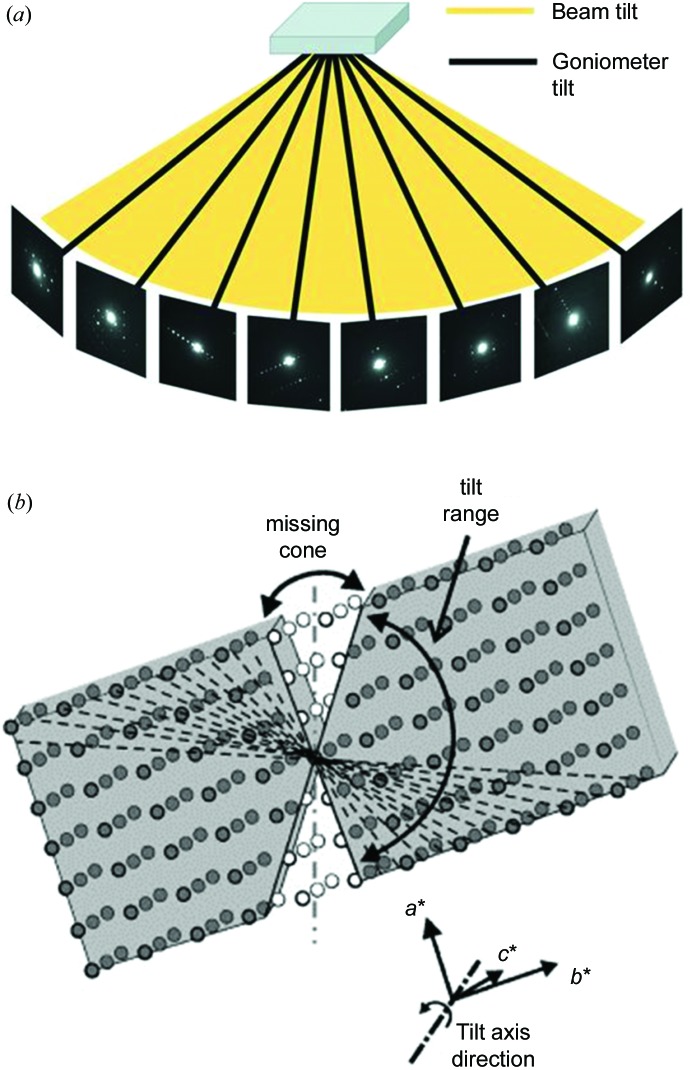
Schematic representations of the concepts of (*a*) the RED method and (*b*) the ADT method. Both can be used to collect ED data by rotating a crystal around an axis in steps of 1–3° using goniometer rotation. To cover the gaps between goniometer rotations, RED uses a fine beam tilt (typically 0.05–0.2°), while ADT is often coupled with precession ED. (*a*) Reprinted (adapted) with permission from Wan *et al.* (2013 ▶). (*b*) Reprinted (adapted) with permission from Mugnaioli & Kolb (2013 ▶). Copyright (2013) Elsevier.

ADT usually uses discrete goniometer tilts in small steps (∼1.0°), with or without continuous precession ED to cover reciprocal space. The data collection can be carried out either in the nano-diffraction mode, together with STEM imaging for tracking crystal movement (Gorelik *et al.*, 2011 ▶) (Fig. 1 ▶ *b*), or *via* selected-area ED with TEM imaging for crystal tracking (Gorelik *et al.*, 2011 ▶; Palatinus *et al.*, 2011 ▶; Gemmi *et al.*, 2012 ▶; Fan *et al.*, 2013 ▶). The *ADT3D* software package is used for ED data processing and ED intensity extraction (Gorelik, Schlitt & Kolb, 2012 ▶).

The RED method combines coarse goniometer tilts (1.0–2.0°) with fine electron beam tilts (0.05–0.50°) on a TEM in selected-area ED or nano-diffraction mode (Fig. 1 ▶). This is similar to the rotation method introduced in single-crystal X-ray diffraction (Arndt & Wonacott, 1977 ▶). RED data collection is controlled using the *RED* data-collection software package (Wan *et al.*, 2013 ▶). Typically, more than 1000 ED frames are collected in about 1 h and used for structure determination. For phase identification *via* determination of the unit cell, only 20–50 ED frames with large steps are needed and the data collection only takes a few minutes. After data collection, the ED frames are processed by the *RED* data-processing software package (Wan *et al.*, 2013 ▶).

Both ADT and RED provide three-dimensional ED data from single crystals of nanometre to submicrometre sizes by goniometer rotation. In ADT, fine sampling of reciprocal space is done by PED, which requires dedicated PED hardware, while in RED this is done by tilting the electron beam, which is controlled by the *RED* software without the need for any hardware. Crystal tracking in ADT uses STEM combined with data acquisition in nano-diffraction mode, which may have an advantage over RED for beam-sensitive materials. Both methods have been applied successfully to the phase identification and structure determination of a variety of materials, as will be shown in the examples given in this review. The data quality is similar, as reflected by the similar *R*_1_ values after structure refinement.

In addition to the advantages of conventional ED, three-dimensional ED methods have many other advantages. Firstly, almost complete three-dimensional ED data can be collected automatically from a nano- or micrometre-sized single crystal. Secondly, since all ED frames in three-dimensional ED data are usually measured off the zone axes, dynamic effects are reduced compared with zonal ED patterns. The intensities from three-dimensional ED data are of good quality and can be used directly for *ab initio* structure solution and further structure refinement using standard structure determination software for X-rays. Data collection can start at any arbitrary orientation of the crystal, which facilitates automation and is easy for non-TEM experts. Three-dimensional ED methods turn a TEM into a single-crystal electron diffractometer and make three-dimensional ED data collection much easier. They have already been shown to be very powerful for the phase identification and structure determination of different types of materials with known or unknown structures.

## 3. Application of three-dimensional ED methods combined with PXRD   

Three-dimensional ED methods and PXRD are complementary to each other. Three-dimensional ED is powerful for phase identification and structure solution from individual nano- or micron-sized particles, while PXRD provides information from all the phases present in the sample. ED suffers from dynamic scattering, while PXRD data is kinematic. For beam-sensitive samples, three-dimensional ED data may have lower resolution and less completeness than PXRD data. Here, the two three-dimensional ED methods, ADT and RED, are described. Examples are given of combinations of the three-dimensional ED methods and PXRD for phase identification and structure determination over a large number of different materials, from Ni–Se–O–Cl crystals, zeolites, germanates, metal–organic frameworks and organic compounds to intermetallics with modulated structures. We show that three-dimensional ED is now as feasible as PXRD for phase identification and SCXRD for structure solution.

### 3.1. Phase identification   

Phase identification is useful for synthetic chemists, mineralogists and metallurgists who are searching for and identifying new compounds when investigating a new chemical system. For many materials, it is not very easy to obtain pure samples in the initial syntheses of a new system. Phase identification of the individual phases from these multiphase powder samples is crucial. Three-dimensional ED methods are used to identify interesting new phases in as-synthesized samples when only polycrystalline powders are available and the samples contain several phases. As a general procedure for phase identification, the crystals are first studied using three-dimensional ED and their unit-cell parameters and symmetries are obtained from the three-dimensional ED data. Next, these parameters are used for profile fitting of PXRD data and indexing of the peaks in the PXRD pattern. If unindexed peaks remain, more effort must be spent on the TEM to find these unidentified phases in the sample. These steps are iterated until all important phases in the sample are found and all strong peaks in the PXRD pattern are indexed. There have been several successful examples of three-dimensional ED methods in the phase identification of multiphase samples in different materials (Birkel *et al.*, 2010 ▶; Gorelik, Sarfraz *et al.*, 2012 ▶; Capitani *et al.*, 2014 ▶; Hua *et al.*, 2014 ▶; Mayence *et al.*, 2014 ▶; Yun *et al.*, 2014 ▶). A few examples are presented below.

**Figure 2:**
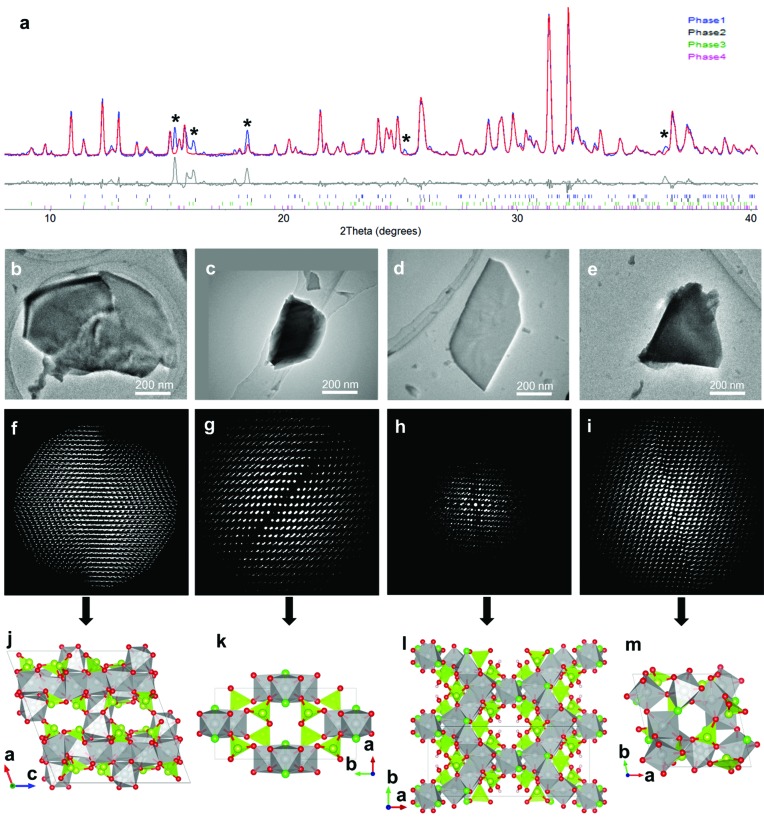
(*a*) Rietveld refinement against the PXRD pattern using the four phases of a multiphase Ni–Se–O–Cl sample determined from RED data (λ = 1.5418 Å). Unindexed peaks are marked by asterisks (*). TEM images showing crystals of (*b*) Phase 1, (*c*) Phase 2, (*d*) Phase 3 and (*e*) Phase 4 used for RED data collection. The corresponding three-dimensional reciprocal lattices of (*f*) Phase 1, (*g*) Phase 2, (*h*) Phase 3 and (*i*) Phase 4 reconstructed from RED data. Structure models of (*j*) Phase 1 (NiSeO_3_), (*k*) Phase 2 (Ni_3_Se_4_O_10_Cl_2_), (*l*) Phase 3 (Ni_5_Se_6_O_16_Cl_4_H_2_) and (*m*) Phase 4 (Ni_5_Se_4_O_12_Cl_2_) determined from RED data. The NiO_4_Cl_2_, NiO_5_Cl and NiO_6_ octahedra are in grey, SeO_3_ trigonal pyramids and Se atoms in yellow green, O atoms in red, Cl atoms in green and H atoms in brown. More information is given by Yun *et al.* (2014 ▶), from where this image is reproduced.

A multiphase Ni–Se–O–Cl sample containing at least four distinct phases was obtained in a search for new compounds (Yun *et al.*, 2014 ▶). The very complex PXRD pattern from the Ni–Se–O–Cl sample (Fig. 2 ▶ *a*) could not be indexed using existing known phases. TEM shows that the crystals in the sample were very small (less than a few micrometres in size) and had different morphologies, indicating that the sample was multiphasic. RED data sets collected on three particles with different morphologies indicated the presence of three different phases (Phases 1–3; Figs. 2 ▶ *b*–2 ▶ *d* and 2 ▶ *f*–2 ▶ *h*). Using the unit-cell parameters and possible space groups deduced for these three phases from the RED data, most of the peaks in the PXRD pattern could now be indexed. The presence of some unindexed peaks suggested that there were still unidentified phase(s) in the sample. Therefore, more effort was spent on the TEM and another phase, named Phase 4, was found (Figs. 2 ▶ *e* and 2 ▶ *i*). While Phase 1 was identified as NiSeO_3_ based on the space group and unit cell, the other three phases could not be found in the ICSD (Inorganic Crystal Structure Database, Version 2013-2; http://icsd.fiz-karlsruhe.de/icsd/), indicating that they were new. With these four phases, there were still a few unindexed weak peaks in the PXRD pattern (Fig. 2 ▶ *a*). Despite further effort spent on the TEM, it was not possible to find more phases corresponding to these peaks. This example shows that three-dimensional ED is powerful for phase identification of polycrystalline samples, especially in identifying unknown phases in multiphase samples.

Another example is PKU-16 {|(C_7_H_10_N_2_)_8_(H_2_O)_12.2_(HF)_5.8_|[Si_0.59_Ge_0.41_O_2_]_64_}, a germano­silicate zeolite with straight three-dimensional 11 × 11 × 12 ring channels (Hua *et al.*, 2014 ▶). PKU-16 was first found as a minor phase in an as-synthesized sample. RED showed that PKU-16 has a tetragonal unit cell with *a* ≃ 19.04 and *c* ≃ 11.73 Å. The possible space groups were *P*4_2_/*mnm, P*4*nm* and 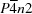, as deduced from the reflection conditions identified from the RED data. The structure was solved from the three-dimensional ED intensities using direct methods with the space group *P*4_2_/*mnm*. The synthesis conditions were then modified in favor of the formation of PKU-16 and pure samples were finally obtained. This example showed that phase identification in multiphase as-synthesized samples using the RED method is very helpful in discovering new interesting structures.

Three-dimensional ED methods can also be used for the phase identification of minerals. Three Bi sulfate phases from the Alfenza Mine, Crodo, Italy, including two new phases and one rare mineral, were identified using ADT (Capitani *et al.*, 2014 ▶). The first new phase, Bi_2_(SO_4_)(OH)_4_, was about 20 µm wide and a few micrometres thick. It has a monoclinic cell [*a* = 22.0 (4), *b* = 16.7 (3) and *c* = 15.9 (3) Å, and β = 102.9 (5)°] with possible space groups *Pc* and *P*2/*c*, which were identified from ADT data. The second new phase, (S_2_)_1+*x*_[Bi_9−*x*_Te_*x*_(OH)_6_O_8_(SO_4_)_2_]_2_, could only be found in TEM due to the small amount present. It has a hexagonal cell [*a* = 9.5 (2) and *c* = 15.4 (3) Å] and space group 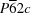. The third phase is cannonite, Bi_2_O(SO_4_)(OH)_2_, with a monoclinic cell [*a* = 7.7 (2), *b* = 13.9 (3) and *c* = 5.7 (1) Å, and β = 109.8 (5)°] and space group *P*2_1_/*c*.

**Figure 3:**
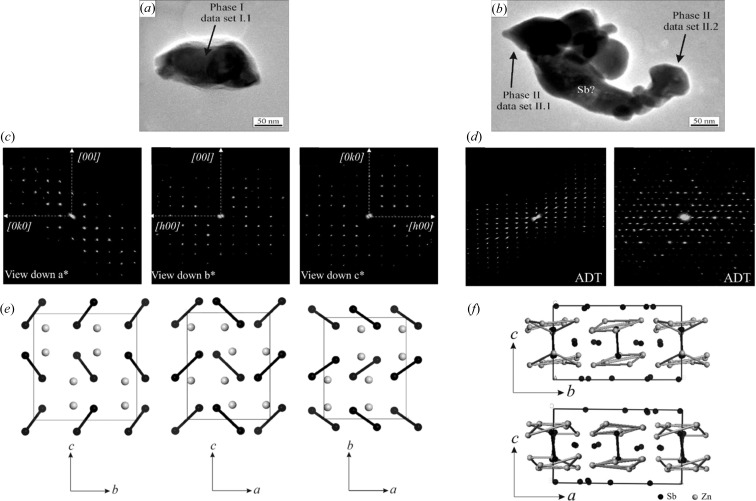
Particles of (*a*) Phase 1 (ZnSb) and (*b*) Phase 2 (Zn_1+δ_Sb) of a new thermoelectric ZnSb system. Projections of the full three-dimensional reciprocal space from ADT for (*c*) Phase 1 and (*d*) Phase 2. Structures of (*e*) Phase 1 and (*f*) Phase 2 determined by *SIR2008*. Reprinted (adapted) with permission from Birkel *et al.* (2010 ▶). Copyright (2010) the American Chemical Society.

Three-dimensional ED was used to study a new thermoelectric ZnSb system (Birkel *et al.*, 2010 ▶). ADT data were collected from different crystals in the sample, which showed unambiguously that the sample consisted of at least two different phases (Fig. 3 ▶). Phase I is a known phase of ZnSb (space group *Pbca*, *a* = 6.54, *b* = 8.06 and *c* = 8.31 Å). Its structure could also be solved by direct methods from the ADT data, and the structure model agreed well with the known crystal structure. The errors in the atomic positions were 0.02 Å for Sb and 0.05 Å for Zn. Phase 2 is a new phase with pseudo-hexagonal symmetry (Zn_1+δ_Sb, space group 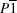, *a* = 15.25, *b* = 15.71 and *c* = 7.81 Å, and α = 90, β = 90 and γ = 120°). Two tilt series of ADT were collected on the same crystal with an in-plane rotation of about 90°. These two data sets were then merged and used for structure solution. The structure of Phase 2 was initially solved in space group *P*1. After examination of the structure model, a higher symmetry was found and the final structure was solved in space group 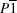 using direct methods. The structure of Phase 2 was solved and refined independently from two ADT data sets from different crystals, with final *R* = 0.362 and 0.425, respectively.

### 3.2. Structure determination   

Intensities obtained by ADT/RED can be used for *ab initio* structure determination of unknown compounds using standard structure determination software for X-rays. Different structure solution methods have been applied to ADT/RED data. These include direct methods [*e.g.* the programs *SHELX* (Sheldrick, 2008 ▶) and *SIR* (Altomare *et al.*, 1999 ▶, 2008 ▶)], charge flipping [*e.g.* the programs *SUPERFLIP* (Palatinus & Chapuis, 2007 ▶) and *JANA* (Petříček *et al.*, 2014 ▶)] and simulated annealing (*e.g.* *SIR*). The structural models can be refined by full-matrix least-squares refinement using *SHELX*. The kinematic approach has been applied, *i.e.* the ED intensities are proportional to the square of the structure factor amplitudes. Atomic scattering factors for electrons are used instead of those for X-rays. Three-dimensional ED intensities are of lower quality than those of X-ray diffraction, largely due to inelastic scattering and dynamic effects, but are good enough for structure determination and refinement. Although refinement against three-dimensional ED data usually converges to structure models with accurate coordinates, the high *R* values prevent convincing structure validation. In order to confirm the structures obtained from three-dimensional ED methods, the structure models are usually refined against PXRD using Rietveld refinement. Both ADT and RED have been used for structure determination of many different types of complex materials. These include four phases in an Ni–Se–O–Cl system (Yun *et al.*, 2014 ▶), zeolites (Jiang *et al.*, 2011 ▶, 2015 ▶; Martínez-Franco *et al.*, 2013 ▶; Mugnaioli & Kolb, 2013 ▶, 2014 ▶; Guo *et al.*, 2014 ▶; Hua *et al.*, 2014 ▶; Lee *et al.*, 2014 ▶; Lorgouilloux *et al.*, 2014 ▶; Su *et al.*, 2014 ▶; Willhammar, Xie, McCusker *et al.*, 2014 ▶), open-framework germanates (Fang *et al.*, 2014 ▶), metal–organic frameworks (MOFs) (Denysenko *et al.*, 2011 ▶; Feyand *et al.*, 2012 ▶; Mugnaioli & Kolb, 2013 ▶; Zhu *et al.*, 2013 ▶; Willhammar, Yun & Zou, 2014 ▶), organic compounds (Kolb *et al.*, 2010 ▶; Gorelik, van de Streek *et al.*, 2012 ▶; Zhang *et al.*, 2013 ▶), intermetallics with modulated structures (Palatinus *et al.*, 2011 ▶; Boullay *et al.*, 2013 ▶; Singh *et al.*, 2014 ▶) and many other structures (Mugnaioli *et al.*, 2009 ▶; Mugnaioli, Andrusenko *et al.*, 2012 ▶; Mugnaioli, Gorelik *et al.*, 2012 ▶; Mugnaioli, Sedlmaier *et al.*, 2012 ▶; Rozhdestvenskaya *et al.*, 2010 ▶, 2011 ▶; Andrusenko *et al.*, 2011 ▶; Gemmi *et al.*, 2011 ▶, 2012 ▶; Kolb *et al.*, 2011 ▶; Sedlmaier *et al.*, 2011 ▶; Bellussi *et al.*, 2012 ▶; Gemmi & Oleynikov, 2013 ▶; Li *et al.*, 2013 ▶; Rius *et al.*, 2013 ▶; Mayence *et al.*, 2014 ▶; Pignatelli *et al.*, 2014 ▶; Plášil *et al.*, 2014 ▶; Roussel *et al.*, 2014 ▶). The RED method has also been combined with HRTEM imaging for structure solution of disordered zeolites (Willhammar *et al.*, 2012 ▶). Three-dimensional ED methods have also been combined with other methods for structure characterization (Schmidt *et al.*, 2009 ▶; Gorelik *et al.*, 2010 ▶; Goian *et al.*, 2012 ▶; Bekheet *et al.*, 2013 ▶; Garcia-Martinez *et al.*, 2014 ▶; Imlau *et al.*, 2014 ▶; Smeets, Xie, Baerlocher *et al.*, 2014 ▶; Xu *et al.*, 2014 ▶; Zhu *et al.*, 2014 ▶). To date, some 200 structures have been identified or solved by three-dimensional ED methods. The structure determinations of a few of the structures mentioned above are described below.

#### 3.2.1. Ni–Se–O–Cl crystals   

The structures of all four phases found as submicron-sized crystals in an Ni–Se–O–Cl system were solved from RED data using direct methods (Yun *et al.*, 2014 ▶) (Fig. 2 ▶). Phase 1 (NiSeO_3_) is a known compound already in the ICSD. It has a monoclinic cell (*a* = 15.58, *b* = 9.96 and *c* = 14.82 Å, and β = 110.2°) in space group *C*2*/c*. All 20 symmetry-independent atoms (four Se, four Ni and 12 O atoms) were located directly in the structure solution. The structure model was refined against RED data (*R*_1_ = 0.295 for 1142 independent reflections). Comparing the corresponding atomic positions of the structure model from RED and those of the structure in the ICSD database, the maximum deviations are 0.05 Å for Ni, 0.05 Å for Se and 0.13 Å for O.

Phase 2 (Ni_3_Se_4_O_10_Cl_2_) is a new compound, isostructural with Co_3_Se_4_O_10_Cl_2_ which was recently solved by single-crystal X-ray diffraction (Rabbani *et al.*, 2014 ▶). It has a monoclinic cell (*a* = 7.17, *b* = 13.70 and *c* = 5.63 Å, and β = 106.8°) in space group *C*2*/m*. All seven symmetry-independent atoms (one Se, two Ni, one Cl and three O atoms) were located directly in the structure solution. The final refinement against RED data without any geometric restraints converged to *R*_1_ = 0.220 for 248 independent reflections. Comparing the atomic positions of Ni_3_Se_4_O_10_Cl_2_ from the RED data and those of Co_3_Se_4_O_10_Cl_2_ from SCXRD, the maximum deviations are 0.11 Å for the O atoms and less than 0.038 Å for the non-oxygen atoms.

Phase 3 [Ni_5_Se_6_O_14_(OH)_2_Cl_4_] is monoclinic (*a* = 21.94, *b* = 8.38 and *c* = 12.68 Å, and β = 118.1°) in space group *C*2*/c*. All 16 symmetry-independent non-hydrogen atoms (three Se, three Ni, two Cl and eight O atoms) were located directly in the structure solution using direct methods. The structure model of Phase 3 was then refined to *R*_1_ = 0.303 for 351 independent reflections.

Phase 4 (Ni_5_Se_4_O_12_Cl_2_) is triclinic (*a* = 9.44, *b* = 9.44 and *c* = 8.14 Å, and α = 105.1, β = 91.6 and γ = 101.6°). A reasonable solution was obtained in space group 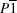. All 23 symmetry-independent atoms (four Se, five Ni, two Cl and 12 O atoms) were located directly in the structure solution using direct methods. The refinement against the RED data of Phase 4 converged to *R*_1_ = 0.405 for 1464 independent reflections.

#### 3.2.2. Zeolites   

**Figure 4:**
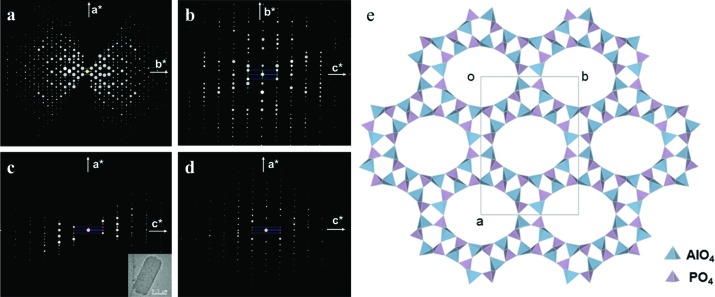
(*a*)–(*d*) Two-dimensional slices of the reciprocal lattice of ITQ-51 reconstructed from three-dimensional RED data. (*a*)–(*c*) Two-dimensional slices from data set 1. The crystal is shown as an inset in (*c*). (*d*) A two-dimensional (*h*0*l*) slice from data set 2, showing that the two data sets cover different parts of reciprocal space. (*e*) A structural model for ITQ-51, viewed along the *c* axis. Reprinted (adapted) with permission from Martínez-Franco *et al.* (2013 ▶). Copyright (2013) the National Academy of Sciences, USA.

Zeolites often form polycrystalline powders and three-dimensional ED methods are well suited to the structure determination of such zeolites. One example is the silico­alumino­phosphate ITQ-51 (Si_2.6_Al_14.7_P_14.7_O_64_), with extra-large 16-ring channels (Martínez-Franco *et al.*, 2013 ▶). Two RED data sets from different ITQ-51 crystals were collected and merged to achieve high completeness (Fig. 4 ▶). The unit-cell parameters [*a* = 23.345 (2), *b* = 16.513 (2) and *c* = 4.9814 (5) Å, and α = 90, β = 90.620 (5) and γ = 90°] were first determined from RED data and further refined against PXRD data. The space group *P*2_1_/*n* was deduced from the reflection conditions identified from the RED data. 2310 independent reflections with a completeness of 81.8% (*d* > 0.9 Å) were obtained from the merged RED data set. All eight symmetry-independent framework *T* (*T* = Al, Si, P) atoms and 16 O atoms could be located by direct methods from the RED data. The structure model was refined against the RED intensities (*R*_1_ = 0.37). The Al and P positions could be identified based on the difference between the Al—O and P—O distances. The structure model was further refined using Rietveld refinement against PXRD data. The atomic positions obtained from RED data (as-synthesized ITQ-51 sample) deviated on average by 0.11 Å for Al/P and 0.13 Å for O from those obtained after the Rietveld refinement (calcined ITQ-51 sample).

**Figure 5:**
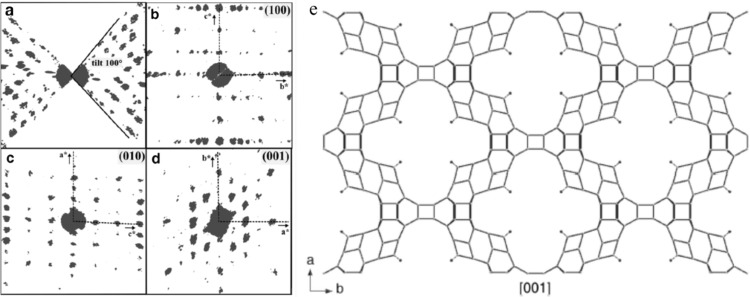
(*a*)–(*d*) Three-dimensional projections of the reconstructed reciprocal lattice of ITQ-43 from ADT data, viewed along (*a*) the tilt axis, (*b*) the a* axis, (*c*) the b* axis and (*d*) the c* axis. (*e*) A structural model for ITQ-43, viewed along the *c* axis. Reprinted with permission from Jiang *et al.* (2011 ▶). Copyright (2011) the American Association for the Advancement of Science.

The above-mentioned zeolite ITQ-51 was relatively stable under the electron beam. It was therefore possible to collect high-quality RED data under normal conditions. For materials that are more sensitive to electron irradiation, a cryo-holder may be beneficial in reducing beam damage, for example in the case of ITQ-43 (Si_0.69_Ge_0.31_O_2_; Fig. 5 ▶; Jiang *et al.*, 2011 ▶). In addition, merging three-dimensional data from different particles can improve data quality for beam-sensitive materials. ITQ-43 (space group *Cmmm*, *a* = 26.090, *b* = 41.866 and *c* = 12.836 Å) is a meso­porous germano­silicate zeolite. ADT data collected at room temperature could only be used for unit-cell determination and not for structure determination, due to the low data quality as a result of beam damage. Cooling the sample improved the stability of the crystals under the electron beam, and high-quality ADT data could be collected. Two ADT data sets were collected from two small particles (dimensions 200 × 90 nm and 250 × 140 nm) at ∼100 K using a cryo-holder and merged for structure determination. All 20 independent Si/Ge positions and 24 of the 42 O positions were found using direct methods, in space group *C*222. Thirteen more O positions were identified during subsequent Fourier refinement. The remaining five O positions were added based on the geometry. The refined structure model showed *Cmmm* symmetry. The final structure model was refined by Rietveld refinement against PXRD data, resulting in final *R*_exp_, *R*_wp_, *R*_F_ and *R*_B_ residuals of 0.042, 0.160, 0.096 and 0.075, respectively.

**Figure 6:**
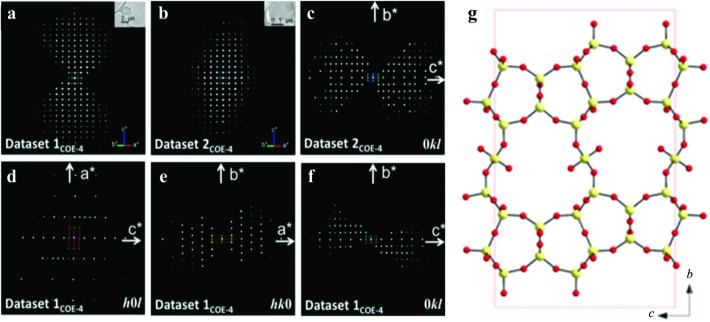
Reconstructed three-dimensional reciprocal lattices of COE-4, (*a*) data set 1_COE-4_ and (*b*) data set 2_COE-4_, taken from two crystals. The crystal size and morphology are shown as insets. (*c*) Two-dimensional 0*kl* slices cut from data set 2_COE-4_. (*d*)–(*f*) Three two-dimensional *h*0*l*, *hk*0 and 0*kl* slices cut from data set 1_COE-4_. (*g*) The structure of the layered silicate COE-4 determined from RED data. Reprinted (adapted) with permission from Guo *et al.* (2014 ▶). Copyright (2014) the Royal Society of Chemistry.

The RED method was used for structure determination of interlayer expanded zeolites, for example the structure determination of silicates COE-3 and COE-4 (Guo *et al.*, 2014 ▶); COE-4 is the calcined form of the as-synthesized COE-3 material. Three RED data sets of COE-3 [Si_39.3_O_74.6_(CH_3_)_6.6_(OH)_1.4_] were collected from crystals with different orientations. The unit cell and possible space groups could be determined (*a* = 7.2, *b* = 22.4 and *c* = 13.6 Å, space groups *Cmc*2_1_, *C*2*cm* or *Cmcm*) from each data set. The structure of COE-3 could be solved only from the merged data set using direct methods in space group *Cmcm*. All five Si atoms, including the bridging Si atom between the ferrierite layers, and five out of eight O atoms in the asymmetric unit could be found. The three missing O atoms were added according to the geometry of the SiO_4_ tetrahedra. The refinement converged to *R*_1_ = 0.38 for 227 observed reflections. The structure of COE-4 [Si_38.7_O_73.4_(OH)_8_, space group *Cmcm*, *a* = 7.3, *b* = 22.0 and *c* = 14.0 Å] was solved using direct methods from a data set merged from two data sets that were complementary in orientations (Fig. 6 ▶). All five Si atoms and six out of nine O atoms in the asymmetric unit were found. The three missing O atoms were added according to the geometry of the SiO_4_ tetrahedra. The refinement converged to *R*_1_ = 0.38 for 359 observed unique reflections. In order to confirm the structure models for COE-3 and COE-4 from RED, COE-3 and COE-4 were refined against PXRD data and the refinements converged to *R*_wp_ = 0.043 for COE-3 and 0.049 for COE-4.

In addition to using direct methods for structure solution from three-dimensional ED data, other methods or software can also be used, for example charge flipping and the zeolite-specific structure solution program *FOCUS* (Smeets *et al.*, 2013 ▶). SSZ-45 is a high-silica zeolite {|(C_11_H_21_N_2_)_8_(OH)_8_|[Si_200_O_400_]; Smeets, Xie, McCusker *et al.*, 2014 ▶}. All eight RED data sets showed that SSZ-45 has an *F*-centred orthorhombic cell, which clarifies the ambiguity from PXRD. The two best RED data sets were merged and used for structure solution. The non-overlapping reflections from laboratory PXRD data were added to the RED data for structure determination using the program *SUPERFLIP* (Palatinus & Chapuis, 2007 ▶). A layered structure was found in the solution. By adding a layer of isolated four-rings between the layers, a structure model for SSZ-45 could be constructed. Structure solution was also achieved using a special version of *FOCUS* modified for electron data. The same structure model was obtained. However, the time needed to find the structure solution was much shorter using three-dimensional ED data than when using PXRD. The structure of SSZ-45 was then refined against PXRD data by Rietveld refinement in the space group *Fmm*2.

#### 3.2.3. Open-framework germanates   

**Figure 7:**
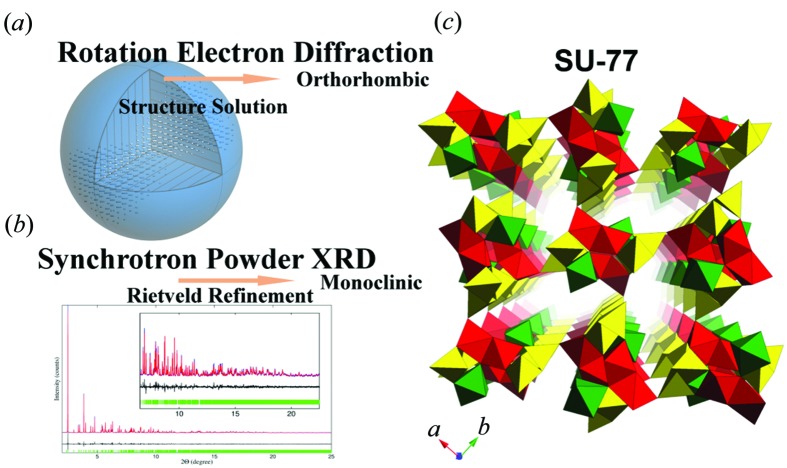
(*a*) Reconstructed RED data from SU-77 used for structure solution, showing an orthorhombic unit cell. (*b*) Rietveld refinement of SU-77 against synchrotron PXRD, showing a monoclinic unit cell. (*c*) Structural model of as-synthesized SU-77, viewed along the [001] direction. Reprinted (adapted) with permission from Fang *et al.* (2014 ▶). Copyright (2014) the American Chemical Society.

Open-framework germanates are germanium oxides with well defined pore structures, built from mixed Ge–O coordination polyhedra. They are often less stable than zeolites. The structure of the open-framework germanate SU-77 {|(C_2_H_10_N_2_)(C_2_H_9_N_2_)_3_(C_2_H_8_N_2_)_4_F|[Ge_24_O_48_F_4_]} was solved and confirmed by combining RED and PXRD (Fang *et al.*, 2014 ▶). Two RED data sets were collected from different crystals. The ortho­rhombic structure (space group *Pnam*, *a* = 14.19, *b* = 12.66 and *c* = 9.52 Å) could be solved from each of the data sets by direct methods. Five symmetry-independent Ge atoms and ten symmetry-independent O atoms were located. The resulting open-framework structure was further refined against the RED data. The refinement converged to *R*_1_ = 0.356, 0.380 and 0.354 for data set 1, data set 2 and the merged data set, respectively. The structure models obtained from the two data sets were similar, with average deviations in the atomic positions of 0.06 Å for Ge and 0.15 Å for the O atoms. However, the PXRD data showed that the as-synthesized SU-77 was monoclinic, which is different from the result obtained from RED. This suggests that SU-77 underwent a structure change from monoclinic to orthorhombic within the TEM. *In situ* PXRD confirmed the structure change of SU-77 after losing some of the water molecules in the pores. A structure model with a monoclinic unit cell [space group *P*2_1_/*a*, *a* = 13.52427 (5), *b* = 12.64862 (5) and *c* = 9.60578 (3) Å, and β = 92.8599 (4)°] was then built based on the orthorhombic structure model obtained from the RED data, and this was successfully refined against the PXRD data. SU-77 is the first example of an open framework with mixed coordination polyhedra to have been solved from ED data (Fig. 7 ▶).

#### 3.2.4. Metal–organic frameworks (MOFs)   

Metal–organic frameworks (MOFs) are compounds consisting of ions/clusters and organic components linked together in different spatial arrangements. Although they are usually less stable than zeolites under an electron beam, three-dimensional ED data could still be collected from MOF crystals for structure determination. A few MOF structures have been solved using both ADT and RED, for example Zn-MOF MFU-4 (Denysenko *et al.*, 2011 ▶), Zn zeolitic imidazolate framework ZIF-7 (Willhammar, Yun & Zou, 2014 ▶), Zr-MOF UiO-66 (Zhu *et al.*, 2013 ▶) and Bi-MOF CAU-7 (Feyand *et al.*, 2012 ▶).

The structure of MFU-4, Zn_5_Cl_4_(BTDD)_3_ {H_2_-BTDD = bis(1*H*-1,2,3-triazolo[4,5-*b*][4′′,5′′*i*])dibenzo[1,4]dioxin}, was solved from ADT data (Denysenko *et al.*, 2011 ▶), collected with and without precession at ∼110 K using a cryo-holder. The space group (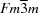) and unit cell (*a* = 31.057 Å) were determined from the ADT data without precession. 412 independent reflections up to a resolution of 1.3 Å were integrated from the ADT data with precession. *Ab initio* structure solution was performed using direct methods. Nine out of the ten unique positions could be located; only one C position was missing. MFU-4 is the first unknown MOF structure solved by electron crystallography.

The organic linkers in MOFs are often known from the synthesis. In order to solve the structure of a MOF, it often suffices to find the unit-cell parameters and symmetry and to locate the metal atoms in the structure. The structure models can easily be completed by adding the linkers. This was the case for the structure solution of ZIF-7 [Zn(benzimidazolate)_2_·3H_2_O]. Even though the RED data of ZIF-7 were collected at ∼90 K using a cryo-holder, the resolution was still low (*d* > 1.43 Å). The unit-cell parameters (*a* = 22.47 and *c* = 15.86 Å) and space group (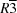) were determined directly from the RED data. Only the Zn and N positions could be located directly using direct methods. Since the linkers were rigid, it was easy to complete the structure model by model building. The structure model could be refined against the RED data using soft restraints on the bond distances and bond angles. The refinement converged to *R*_1_ = 0.28.

**Figure 8:**
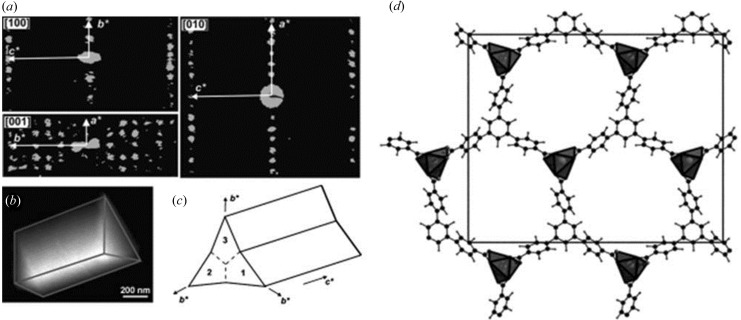
(*a*) Reconstructed three-dimensional diffraction space of CAU-7, projected along the main axes. (*b*) A rod tilted to expose the triangular base and (*c*) a sketch of the trilling arrangement. (*d*) The refined structural model of CAU-7. Reproduced with permission from Feyand *et al.* (2012 ▶). Copyright (2012) John Wiley and Sons.

Twinning in structures does not impose severe difficulties in structure solution from three-dimensional ED data. An example is demonstrated by CAU-7, a Bi-based MOF [Bi(BTB), BTB = 1,3,5-benzenetrisbenzoate; Fig. 8 ▶; Feyand *et al.*, 2012 ▶]. ADT data were collected at 120 K using a cryo-holder. The ADT data showed that each CAU-7 crystal was a twin aggregate of three individuals that grew following the pseudo­hexagonal symmetry of the structure. The unit-cell parameters (*a* = 32, *b* = 28 and *c* = 4 Å) and extinction group (*Pb–a*) were obtained from the ADT data. ADT data sets from two different single twin domains were merged and 1158 independent reflections were obtained to a resolution of 1.15 Å. Only the Bi atom could be located from the ADT ED intensities using direct methods. Simulated annealing (SA) was used for structure solution instead. One Bi atom and one BTB were input in the unit cell. A reasonable structure solution was obtained based on the ADT ED intensities. Finally, the structure model was refined by Rietveld refinement against the PXRD data. A DFT-based calculation was performed to confirm the model.

#### 3.2.5. Organic compounds   

**Figure 9:**
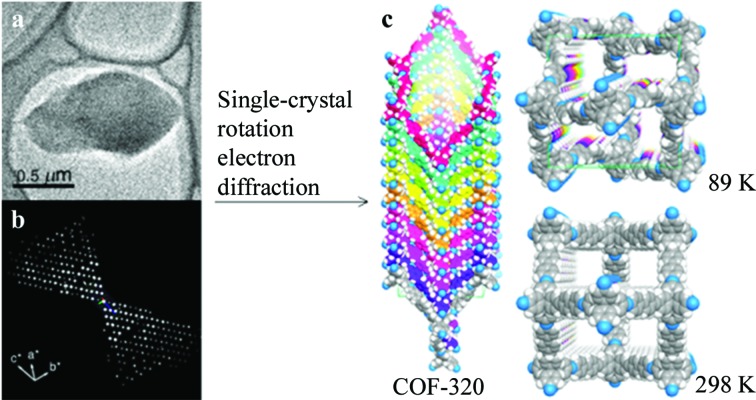
(*a*) The COF-320 particle used for RED data collection and (*b*) the reconstructed three-dimensional RED data. (*c*) Models of COF-320. Reprinted (adapted) with permission from Zhang *et al.* (2013 ▶). Copyright (2013) the American Chemical Society.

Organic compounds are usually very sensitive to an electron beam and collecting complete three-dimensional ED data can be challenging. Some covalent organic frameworks (COFs) are sufficiently stable and allow reasonably complete three-dimensional data to be collected. The structure of a new COF, named COF-320 (C_53_H_36_N_4_), was determined using the RED method (Fig. 9 ▶, Zhang *et al.*, 2013 ▶). RED data sets were collected at both 298 and 89 K. At lower temperatures, less beam damage was observed for COF-320. The RED data set collected at 89 K had a resolution up to 1.5 Å. The unit cell (*a* = 30.17 and *c* = 7.28 Å, and *V* = 6628 Å^3^) and possible space groups, *I*4_1_ *md* and 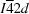, were determined from the RED data. The SA parallel tempering algorithm in *FOX* (Favre-Nicolin & Černý, 2002 ▶) was used to find an initial molecular arrangement from the RED data. The structure of COF-320 was solved in space group 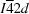 and refined using *SHELXL*. The final refinement converged to *R*_1_ = 0.31. The RED data set collected at 298 K suggested a body-centred orthorhombic unit cell (*a* = 27.93, *b* = 31.31 and *c* = 7.89 Å, and *V* = 6899 Å^3^) and space group *Imma*. Due to the poor quality of this RED data, only the central C atom of the tetrahedral building blocks could be located by direct methods. A model was built based on the crystallographic information from the RED data. The PXRD pattern of an activated sample of COF-320 was in agreement with the pattern calculated from the model at 298 K.

**Figure 10:**
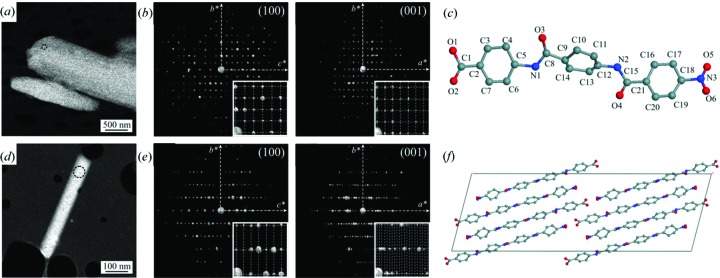
Crystals of (*a*) OPBA3 and (*d*) OPBA4. Views of the reciprocal volume along the main zone axes of (*b*) OPBA3 and (*e*) OPBA4 obtained from ADT. Structures of (*c*) OPBA3 and (*d*) OPBA4 from ADT data using *SIR*. Reprinted (adapted) from Gorelik, van de Streek *et al.* (2012 ▶).

ADT has successfully been used to solve the structures of a number of organics (Kolb *et al.*, 2010 ▶). For the structure determination of oligo-*p*-benzamides (Gorelik, van de Streek *et al.*, 2012 ▶), ADT data were first used to validate the known structure of a tri-*p*-benzamide (OPBA3) by direct methods. ADT was then applied to an unknown structure of a tetra-*p*-benzamide (OPBA4) (Fig. 10 ▶). OPBA3 has a monoclinic cell (*a* = 14.70, *b* = 9.88 and *c* = 12.68 Å, and β = 107.08°) in space group *P*2_1_/*c*. The structure was solved from ADT data and the final refinement converged to *R*_1_ = 0.58. The refined structure is very similar to the one reported. The OPBA4 crystals are needle-like with a monoclinic cell (*a* = 50.77, *b* = 5.32 and *c* = 17.27 Å, and β = 103.24°) and possible space groups *C*2/*c* and *Cc*. The structure of OPBA4 was solved using ADT data by direct methods in space group *C*2/*c*. All atoms except one O atom were located directly in the structure solution. The final refinement was carried out using energy minimization.

#### 3.2.6. Intermetallics with modulated structures   

**Figure 11:**
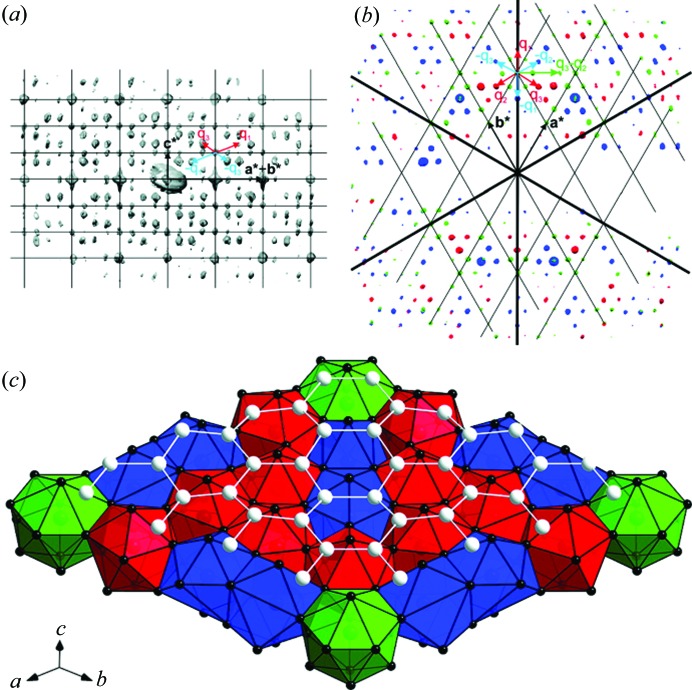
(*a*, *b*) Three-dimensional distributions of diffraction intensities of η′-Cu_3+*x*_(Si,Ge) in reciprocal space. (*a*) A projection along a*–b*, with the basic unit cell and modulation vectors indicated. (*b*) Three layers viewed along c*. (*c*) The structure in the supercell approximation, showing one slab of Cu clusters and one layer of Si/Ge atoms. Reprinted (adapted) with permission from Palatinus *et al.* (2011 ▶). Copyright (2011) the American Chemical Society.

Three-dimensional ED methods can be used for structure determination of rather complex structures, for example incommensurately modulated structures. The structure of η′-Cu_3+*x*_(Si,Ge) was solved from three-dimensional ED data collected from a submicrometre-sized crystal using the charge-flipping algorithm in superspace (Palatinus *et al.*, 2011 ▶). The structure has a trigonal cell and it is incommensurately modulated, with two modulation vectors *q*_1_ = (α, α, 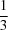) and *q*_2_ = (−2α, α, 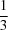) in superspace group 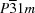
(α, α, 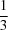)000(−2α, α, 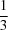)000. The modulated structure can be described as sheets of Cu clusters separated by honeycomb layers of mixed Si/Ge positions. The shape of the Cu clusters in the sheets varies strongly with the modulation phase, and the predominant form is an icosahedron. The striving of the Cu layers to form icosahedral clusters is deemed to be the main driving force of the modulation (Fig. 11 ▶).

**Figure 12:**
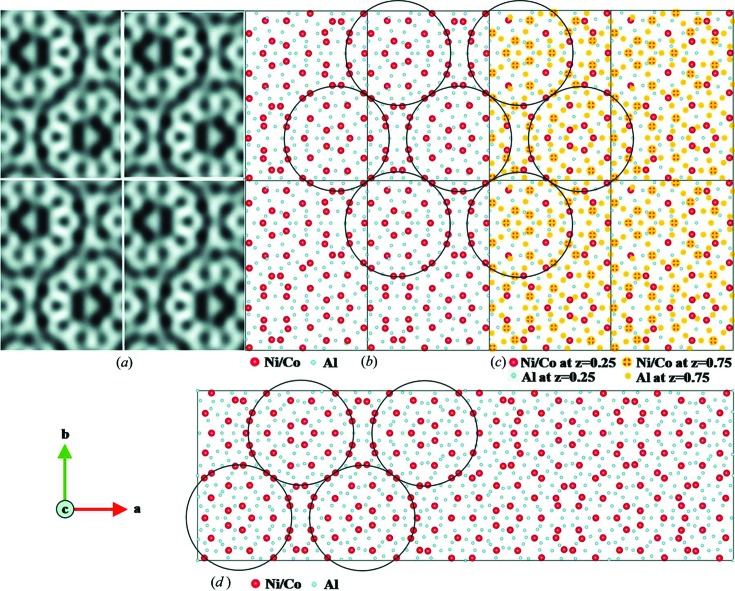
(*a*) HRTEM images of PD2 with 2 × 2 unit cells, taken along the *c* axis (Hovmöller *et al.*, 2012 ▶). The tenfold wheels with atoms in black are clearly seen. (*b*) Atomic structure model of PD2 obtained from RED data, projected along the *c* axis. The circles indicate the 2 nm cluster columns with a pseudo-tenfold rotational symmetry. Ni/Co atoms are in red and Al atoms in blue. (*c*) Structure model showing the arrangement of Ni/Co atoms at *z* = 0.25 (red) and *z* = 0.75 (red with yellow cross) layers. (*d*) The structure of PD4 (*a* = 101.3, *b* = 32.0 and *c* = 4.1 Å), as determined by X-ray crystallography (Oleynikov *et al.*, 2006 ▶). The circles mark the 2 nm clusters similar to those found in PD2. Whole figure reprinted from Singh *et al.* (2014 ▶).

A pseudo-decagonal (PD) quasicrystal approximant Al_37.0_(Co,Ni)_15.5_, also called PD2, was solved using the RED method (Singh *et al.*, 2014 ▶). PD2 has an *F*-centred ortho­rhombic cell with *a* = 46.4, *b* = 64.6 and *c* = 8.2 Å, as determined from RED data. However, the reflections with odd *l* indices were much weaker than those with *l* even. Therefore, the basic structure of PD2 was solved with unit-cell parameters *a* = 23.2, *b* = 32.3 and *c* = 4.1 Å in space group *Pnmm* using direct methods. The basic structure contains 55 unique atoms (17 Co/Ni and 38 Al) and is one of the most complex structures solved by ED to date. The structure was refined against three-dimensional RED data with *R*_1_ = 0.43 for 1799 unique reflections. An HRTEM image and ED patterns calculated from the structure model were used to confirm the structure model from RED data. The structure of PD2 has features that are quite similar to that of PD4, which was obtained from SCXRD (Fig. 12 ▶).

## 4. Discussion and perspectives   

The combination of three-dimensional ED with PXRD provides advantages from both techniques as they are complementary. Examples given in this review have shown that the combination of the two techniques is very powerful for phase identification from multiphase samples and complete structure determination of nano- or micron-sized crystals. We expect this strategy to be increasingly important in the identification and structure determination of new materials which would be difficult using other methods, for example X-ray diffraction, alone.

Three-dimensional ED is essentially single-crystal ED, but from smaller crystals. However, at present it needs to be combined with PXRD for a complete structure determination which includes structure solution and reliable refinement. There are a few reasons why three-dimensional ED alone is not yet adequate for complete structure determination. Although unit-cell parameters can be determined from three-dimensional ED data quickly and unambiguously compared with PXRD, they are less accurate (±2%) than those provided by PXRD. Errors in the unit-cell parameters may lead to ambiguous identification of the crystal symmetry. At present, most three-dimensional ED methods use reflection difference vectors to determine the unit cell. The accuracy of the determination thus depends on all the factors that affect the accuracy of the reconstructed positions of reflections in reciprocal space, including the accuracy in camera length, goniometer rotation, rotation axis determination and two-dimensional reflection position determination. In order to improve the accuracy in unit-cell determination, these factors need to be carefully taken into consideration and methods for handling the errors need to be developed.

We have shown that three-dimensional ED methods are powerful for successful structure solution of nano- and submicrometre-sized crystals. However, there are still cases where the methods may fail, for example crystals that are too thick or too small, highly electron-beam sensitive samples and crystals containing disorder. Thick crystals cause strong dynamic effects and thus low-quality intensities in the ED data, which may not lead to successful structure solution. The size limit on crystals depends on the sample composition and density, as well as on the crystal structure itself. In general, dynamic effects are weaker for crystals containing lighter elements and with less-dense structures. As demonstrated by the examples given in this review, structure solution from three-dimensional ED data is successful for crystals with sizes of up to *ca* 1 × 1 × 1 µm. On the other hand, when crystals are too small (< 10 × 10 × 10 nm), the number of unit cells may not be large enough to give good ED data. Most examples of successful structure solution from three-dimensional ED data have been from crystals of a few hundred nanometres in size. Another factor that prevents successful structure solution is electron-beam damage of the samples, for example zeolites, MOFs, organic crystals and proteins. Electron-beam damage leads to data of low completeness, low resolution and low consistency (high internal *R* values). Disorder in crystals contributes to diffuse scattering in three-dimensional ED data. It is difficult to obtain accurate intensities from such crystals, and conventional structure solution methods cannot be applied to disordered crystals. The problems described above are common to both ADT and RED data.

In most cases, three-dimensional ED intensities are of sufficient quality for structure solution and most often allow structure refinement. However, there are errors in the intensities from various sources, including the geometry and limited sampling points used for data collection (with or without precession or beam tilt), dynamic interactions between the electrons and matter, inelastic scattering of the electrons, and the data-recording cameras. In almost all cases, structure models from three-dimensional ED data need to be refined against PXRD data if available. Errors in the intensities need to be reduced before a reliable structure validation and refinement can be reached using three-dimensional ED data. To deal with dynamic effects in electron scattering, Palatinus and co-workers developed an algorithm to include the calculation of dynamic intensities in the refinement against precession ED data and showed that better structure models and lower *R* values could be obtained (Palatinus *et al.*, 2013 ▶). In order to achieve a more accurate structure model and improve the *R* values, a full dynamic structure refinement against three-dimensional ED data is necessary. However, this is rather demanding and requires well shaped crystals of uniform thickness and an accurate estimation of the crystal orientation at each tilt angle.

Data completeness is crucial for successful structure solution from three-dimensional ED data. Three-dimensional ED methods facilitate automated ED data collection and can be used to collect almost complete three-dimensional data. In a TEM, a maximum tilt range of 145° can be covered using a tomography tilt holder. For structures with high symmetries, reflections in the missing wedges can be complemented using the symmetry operations, while merging different data sets may be necessary to obtain a more complete data set for crystals with low symmetries. A dual-axis tomography holder can be used to collect three-dimensional ED data from different orientations of the same crystal (Birkel *et al.*, 2010 ▶). These data sets can then be merged for further analysis. Three-dimensional ED data can also be collected from crystals with different orientations and merged to give more complete data (Jiang *et al.*, 2011 ▶; Martínez-Franco *et al.*, 2013 ▶; Guo *et al.*, 2014 ▶). For crystals with preferred orientations, for example needle-like and plate-like crystals, it is often difficult to obtain three-dimensional ED data with high completeness, even when data-merging procedures are used. In these cases, sample preparation using an ultramicrotome may be helpful.

Three-dimensional ED methods have also been shown to be successful in the structure determination of relatively electron-beam sensitive crystallites, such as organic compounds. For samples that are not very stable under an electron beam, data collection needs to be carried out in low-dose conditions. Sample cooling using cryo-holders is useful for some materials, depending on the dominant mechanism of damage. To improve data completeness for beam-sensitive materials, data sets collected from several crystals can be merged.

The newly developed three-dimensional ED methods have been shown to be very successful in phase identification from multiphase samples and structure characterization of nano- or micron-sized crystals, which is difficult for conventional XRD methods. Three-dimensional ED methods can be used for the structure determination of a wide range of materials, from simple to complex structures, from inorganic through metal–organic to organic compounds. Three-dimensional ED is now as feasible as XRD for phase identification and structure solution, but still needs further development in order to be as accurate as XRD. Today, the best approach is to combine three-dimensional ED and PXRD. We expect that three-dimensional ED methods will become more and more important for phase identification and structure solution of complex materials in the near future.

## Figures and Tables

**Table 1:** Comparison of SCXRD, PXRD, HRTEM, and two- and three-dimensional ED

	SCXRD	PXRD	HRTEM	ED	
	Three-dimensional	One-dimensional	Two-dimensional	Two-dimensional	Three-dimensional
Crystal size	>5m	>50nm	>5nm	>50nm	>50nm
Unit-cell determination	Easy	Difficult	Requires expertise	Requires expertise	Easy
Symmetry determination	Easy	Difficult	Easy	Requires expertise	Easy
Peak overlap	No	Yes	No	No	No
Data completeness	High	High	Low	Low	High
Intensities	Kinematic	Kinematic	Affected by objective lens	Dynamic	Dynamic
Structure factor phase information	No	No	Yes	No	No
Data collection	Easy	Easy	Requires expertise	Requires expertise	Easy
Structure determination	Easy	Difficult	Requires expertise	Difficult	Easy
Sample information	Individual	Representative	Individual	Individual	Individual
